# Familial *STAG2* germline mutation defines a new human cohesinopathy

**DOI:** 10.1038/s41525-017-0009-4

**Published:** 2017-03-20

**Authors:** Fernanda C. Soardi, Alice Machado-Silva, Natália D. Linhares, Ge Zheng, Qianhui Qu, Heloísa B. Pena, Thaís M. M. Martins, Helaine G. S. Vieira, Núbia B. Pereira, Raquel C. Melo-Minardi, Carolina C. Gomes, Ricardo S. Gomez, Dawidson A. Gomes, Douglas E. V. Pires, David B. Ascher, Hongtao Yu, Sérgio D. J. Pena

**Affiliations:** 1 GENE—Núcleo de Genética Médica, Belo Horizonte, MG Brazil; 2 Departamento de Bioquímica e Imunologia, Instituto de Ciências Biológicas, Universidade Federal de Minas Gerais, Belo Horizonte, MG Brazil; 3 Faculdade de Medicina, Laboratório de Genômica Clínica, Universidade Federal de Minas Gerais, Belo Horizonte, MG Brazil; 4 Department of Pharmacology, Howard Hughes Medical Institute, University of Texas Southwestern Medical Center, Dallas, TX USA; 5 Departamento de Patologia Geral, Instituto de Ciências Biológicas, Universidade Federal de Minas Gerais, Belo Horizonte, MG Brazil; 6 Departamento de Ciência da Computação, Instituto de Ciências Exatas, Universidade Federal de Minas Gerais, Belo Horizonte, MG Brazil; 7 Departamento de Clínica, Patologia e Cirurgia Odontológicas, Faculdade de Odontologia, Universidade Federal de Minas Gerais, Belo Horizonte, MG Brazil; 8 Centro de Pesquisas René Rachou, Fundação Oswaldo Cruz, Belo Horizonte, MG Brazil; 9 Department of Biochemistry, University of Cambridge, Cambridge, UK; 10 Department of Biochemistry, University of Melbourne, Victoria, Australia

## Abstract

We characterize a novel human cohesinopathy originated from a familial germline mutation of the gene encoding the cohesin subunit STAG2, which we propose to call *STAG2*-related X-linked Intellectual Deficiency. Five individuals carry a *STAG2* p.Ser327Asn (c.980 G > A) variant that perfectly cosegregates with a phenotype of syndromic mental retardation in a characteristic X-linked recessive pattern. Although patient-derived cells did not show overt sister-chromatid cohesion defects, they exhibited altered cell cycle profiles and gene expression patterns that were consistent with cohesin deficiency. The protein level of STAG2 in patient cells was normal. Interestingly, STAG2 S327 is located at a conserved site crucial for binding to SCC1 and cohesin regulators. When expressed in human cells, the STAG2 p.Ser327Asn mutant is defective in binding to SCC1 and other cohesin subunits and regulators. Thus, decreased amount of intact cohesin likely underlies the phenotypes of *STAG2*-SXLID. Intriguingly, recombinant STAG2 p.Ser327Asn binds normally to SCC1, WAPL, and SGO1 in vitro, suggesting the existence of unknown in vivo mechanisms that regulate the interaction between STAG2 and SCC1.

## Intellectual disability: mutation in cell cycle protein causes developmental disease

A newly discovered developmental disease is characterized by mutations in a subunit of the cohesin protein involved in cell division. A team led by Sérgio Pena from GENE—Núcleo de Genética Médica, Brazil, and Hongtao Yu from the University of Texas Southwestern Medical Center, USA, describe a Brazilian family with five male relatives, all with intellectual deficiency, short stature, and other abnormalities. The family tree pointed toward an X-linked pattern of inheritance, so the researchers performed a network analysis of 24 genes on the X chromosome known to contribute to mental retardation. They found that all five individuals had a mutation in a gene called *STAG2*, which encodes a subunit of cohesin. The mutant STAG2 did not bind properly to other cohesin subunits in human cells, and patient-derived cells exhibited altered cell cycle profiles. The researchers propose calling the disease “*STAG2*-related X-linked intellectual deficiency”.

## Introduction

The cohesin complex has a central role in the cell biology of eukaryotes.^1–4^ It forms a ring to entrap topologically chromosomes, thereby regulating chromosome folding, transcription, DNA repair, and sister-chromatid cohesion.^3, 5–7^ In human somatic cells, cohesin consists of four core subunits: SMC1, SMC3, SCC1, and one of two related helical repeat proteins STAG1 or STAG2 (Fig. S1). Additional regulators that associate with or modify this cohesin core post-translationally promote or suppress its dynamic binding to chromosomes. A complex between two proteins NIPBL (also known as SCC2) and MAU2 (also called SCC4) is required for loading cohesin properly onto chromatin.^8–10^ A complex between PDS5 and WAPL promotes cohesin release from chromosomes.^11–13^ The ATPase domain of SMC3 undergoes reversible acetylation: it can be acetylated by the ESCO1 and ESCO2 acetyltransferases and deacetylated by HDAC8.^14–19^ This reversible acetylation of SMC3 enables the binding of SORORIN to PDS5.^20^ SORORIN competes with WAPL for binding to PDS5 and stabilizes cohesin on chromosomes.^20, 21^ Moreover, SGO1 binds directly to STAG2 and further shields cohesin from WAPL^22, 23^ protecting sister-chromatid cohesion during mitosis, particularly at centromeres.

Mutations of cohesin and regulators have been linked to human developmental diseases, collectively called cohesinopathies.^7, 24, 25^ There are three known major classes of cohesinopathies, each with distinct phenotypes (Fig. S1). The first class is Roberts Syndrome/SC Phocomelia, caused by pathogenic mutations in *ESCO2* (see RS in Fig. S1). The second class is Cornelia de Lange Syndrome, which can be caused with varying degrees of severity by pathogenic mutations in *NIPBL* (CdLS1), *SMC1* (CdLS2), *SMC3* (CdLS3), *SCC1* (CdLS4), and *HDAC8* (CdLS5). The third class, termed Chronic Atrial and Intestinal Dysrhythmia, affects heart and gut rhythm and is caused by germline mutations in *SGO1*.^26^

While somatic mutations of *STAG2* have been frequently observed in several types of human cancers^27, 28^ and increased *STAG2* dosage has been recently linked to intellectual disability,^29^ no pathogenic germline variant of the X-linked *STAG2* gene has been previously described in humans. Here, we describe an X-linked pedigree with five individuals carrying a p.Ser327Asn (c.980 G > A) mutation in *STAG2*.

## Results

**Fig. 1:**
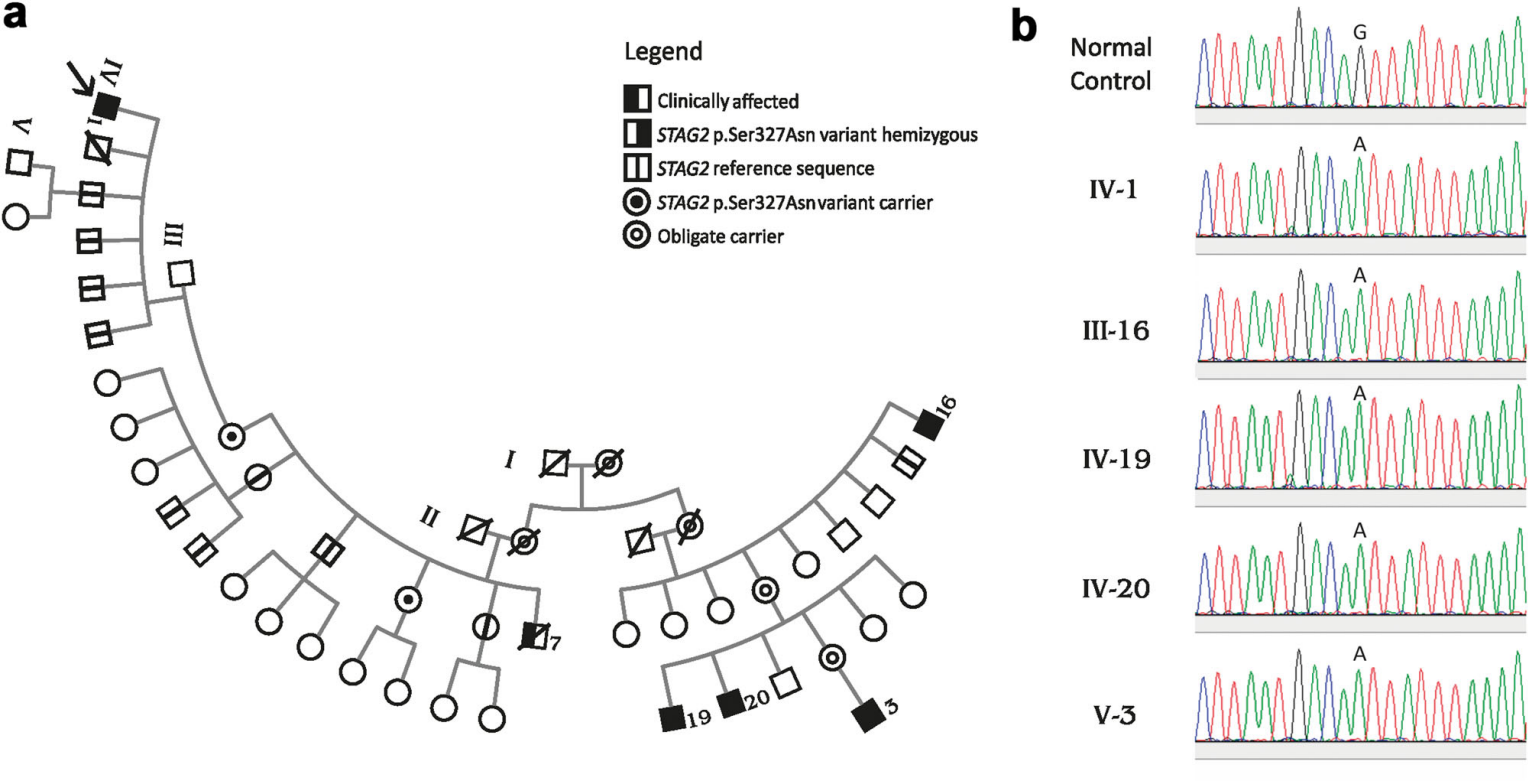
Pedigree. **a** Pedigree shows typical X-linked recessive inheritance of the c.980 G > A (p.Ser327Asn) in the *STAG2* gene (NM_001042749.1) on the X chromosome. The variant was studied in the proband (*arrow*) and in male and female relatives by allele-specific PCR and confirmed in the proband and all affected male relatives by Sanger sequencing. **b** For Sanger sequencing we used the BigDye Terminator v3.1 Cycle Sequencing Kit (Applied Biosystems^®^) and the Applied Biosystems (ABI) 3130 Genetic Analyzer. Sequencing data were analyzed using the software Sequencher version 4.1.4 (Gene Code Corporation)

The proband (individual IV-1 in the pedigree; Fig. 1a) was first seen at age 29, with a clinical picture of moderate intellectual deficiency, short stature, peculiar facies, cleft palate, and unilateral deafness (Supplementary Table 1). A deceased maternal uncle (individual III-7 in Fig. 1a) apparently had a very similar phenotype (Supplementary Table 1), and had died at age 39 supposedly of unrelated causes (Chagas cardiomyopathy). We performed whole exome sequencing (WES) on the proband and we used phenotype-driven variant priorization. First, we tested the variant call format against a list of 562 genes associated with Intellectual Deficiency. No good candidates emerged. Since the history of the deceased maternal uncle suggested the possibility of X-linked inheritance, we used the software Exome Walker to analyze possible candidates interacting with 24 seed genes involved in X-linked mental retardation, provided by the program. The software picked, as first choice, the hemizygous variant c.980 G > A (p.Ser327Asn) in the *STAG2* gene (NM_001042749.1) on the X chromosome. The presence of the variant in the proband was confirmed with allele-specific polymerase chain reaction (PCR) (not shown) and Sanger sequencing (Fig. 1). This variant had in silico pathogenic characteristics as assessed by the prediction programs SIFT (“deleterious”; score = 0.02), PolyPhen-2 (“probably damaging”; score = 0.974), and Mutation Taster (“disease causing”; *p*-value = 1). The c.980 G > A variant was not present in the ExAC database and could not be found in 200 normal males and 100 normal females (400 X chromosomes) from the Brazilian population (not shown). Nor was it found in 200 exomes from Brazilian individuals. We further demonstrated that the mother and an aunt of the proband (III-2 and III-5 in the pedigree; Fig. 1a) were healthy carriers of the c.980 G > A variant. Four brothers of the proband (IV-3-6), one maternal uncle (III-4), two maternal aunts (III-3 and III-6), and two male cousins (IV-10 and IV-11; sons of III-3), all healthy, did not have the c.980 G > A variant, in perfect concordance with a model of X-linked recessive inheritance.

Further family investigations revealed the existence of four distant male maternal relatives with intellectual deficiency and short stature, in a pedigree distribution characteristic of X-linked recessive inheritance (individuals III-16, IV-19, IV-20, and V-3 in the pedigree; Fig. 1a). Allele-specific PCR (not shown) and Sanger sequencing (Fig. 1b) demonstrated that these four individuals were indeed hemizygous for the c.980 G > A (p.Ser327Asn) variant. The healthy brother of one affected individual (III-15) did not carry the variant.

Examination of the five affected individuals and historical information about the deceased one permitted the definition of a consensus phenotype, consisting of moderate intellectual deficiency, short stature, sensory hearing deficiency, large nose, prominent ears, and frontal baldness (Supplementary Table 1). This phenotype defines a new cohesinopathy that we propose to call “*STAG2*-related X-linked Intellectual Deficiency” (Fig. S1).

**Fig. 2:**
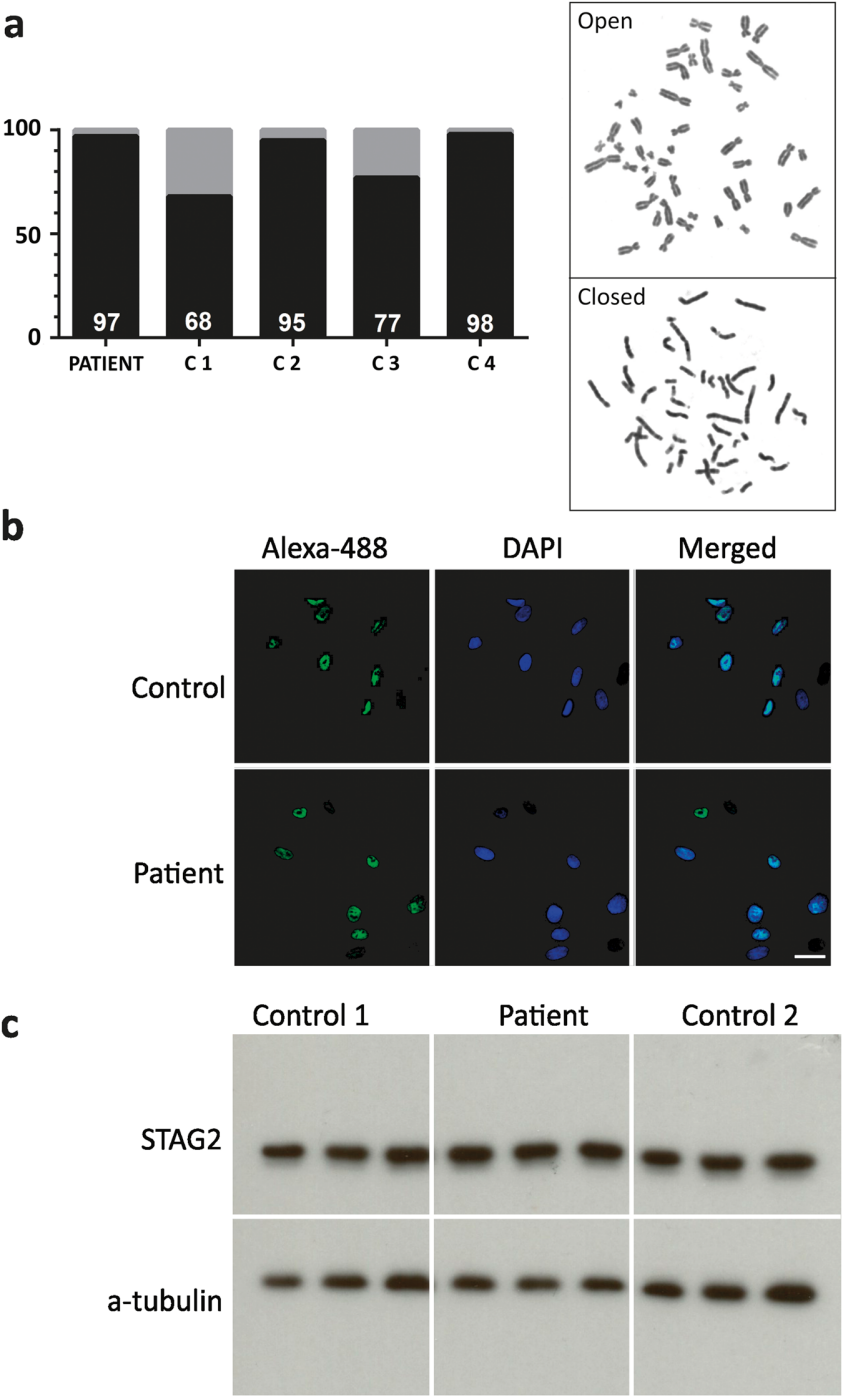
Sister chromatid cohesion assay and immunoreaction experiments. **a** p.Ser327Asn STAG2 does not show effect on the sister chromatid phenotype. Open (*gray*) and closed (*black*) sister chromatids are shown for the patient and four controls with WT STAG2 (C1-4). A total of 100 metaphases were analyzed for each individual. The amount of closed sister chromatids are indicated at the bottom of each individual column. Open and closed sister chromatid phenotypes in metaphase cells from the patient are demonstrated. **b** Immunofluorescence of patient fibroblasts. Cultured fibroblasts from the proband were incubated overnight with anti-STAG2 (J-12) at 4 °C followed by Alexa 488-conjugated second antibody (*green*) and nuclear counterstain with DAPI (*blue*). The nuclear localization of STAG2 was confirmed in the double label immunofluorescence merged picture. Similar results were observed in three independent experiments. **c** Western blot of STAG2 from patient fibroblasts. Protein extracts from patient and control individuals were subjected to immunoblotting using the anti-STAG2 (J-12). After visualization of STAG2, the blot membrane was stripped and re-probed with alpha-tubulin antibody, which was used as a quantitative control. Similar results were observed between the patient and the two controls in three independent experiments

Conventional chromosome analysis of short-term lymphocyte cultures of the proband was normal. We studied the cohesion of sister chromatids by observing the proportion of cells with chromosome arms open and closed at metaphase (Fig. 2a). The proportion of patient cells did not differ from that of normal controls. Immunofluorescence of skin fibroblasts cultures showed the presence of immunoreactive STAG2 in its usual nuclear localization (Fig. 2b). Western blot analysis confirmed that the STAG2 protein was present in normal amounts (Fig. 2c).

**Fig. 3:**
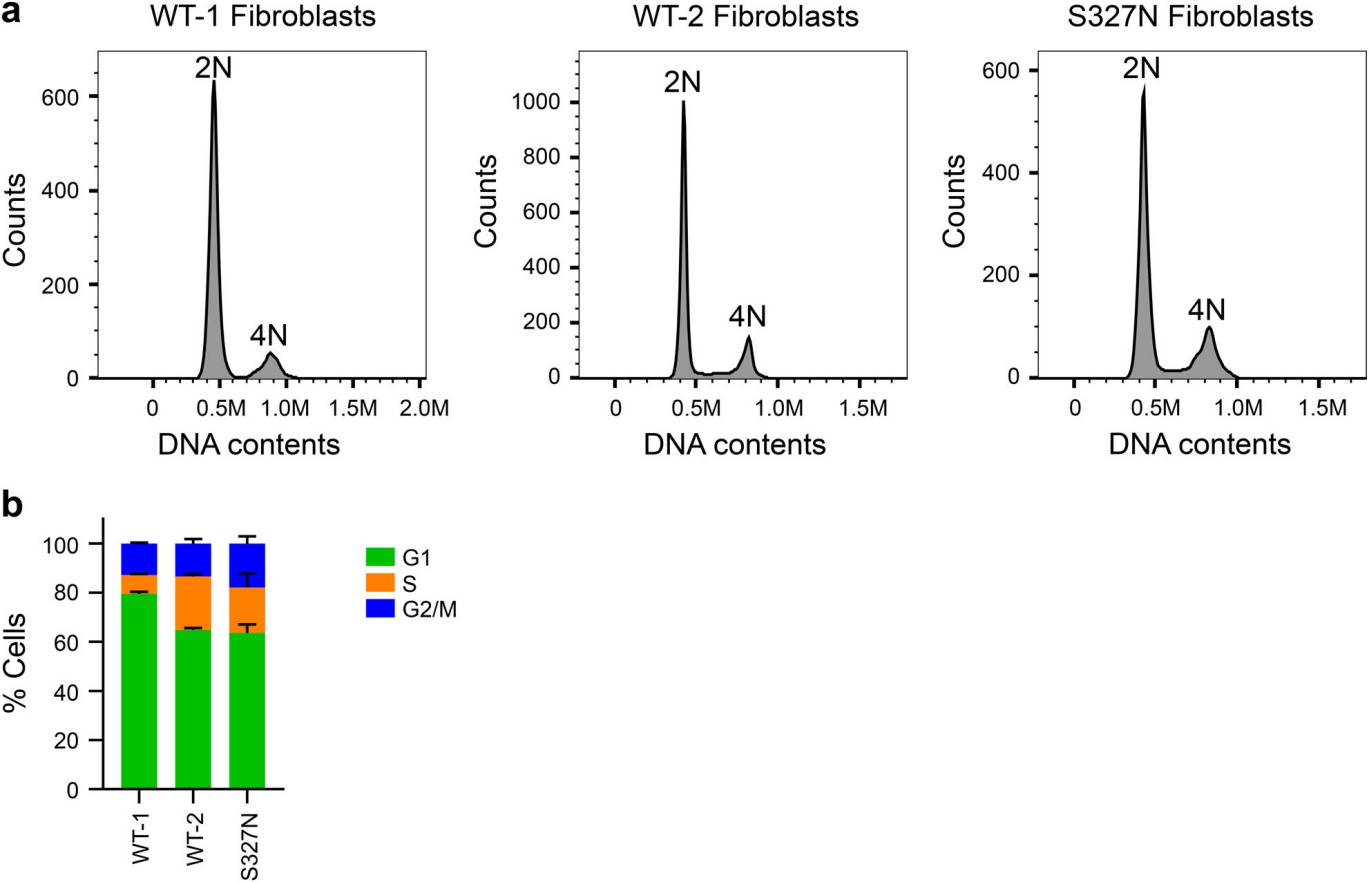
Cell cycle profiles of STAG2 WT and Ser327Asn fibroblasts. **a** Two independent lines of WT fibroblasts (WT-1 and WT-2) and STAG2 Ser327Asn fibroblasts were cultured and stained with propidium iodide and anti-MPM2 antibody. Cells were harvested and analyzed by flow cytometry. Representative graphs are shown, with the cell populations with 2N and 4N DNA contents indicated. **b** Quantification of the fibroblasts in G1, S, and G2/M in **a** (*n* = 2 independent experiments)

**Fig. 4:**
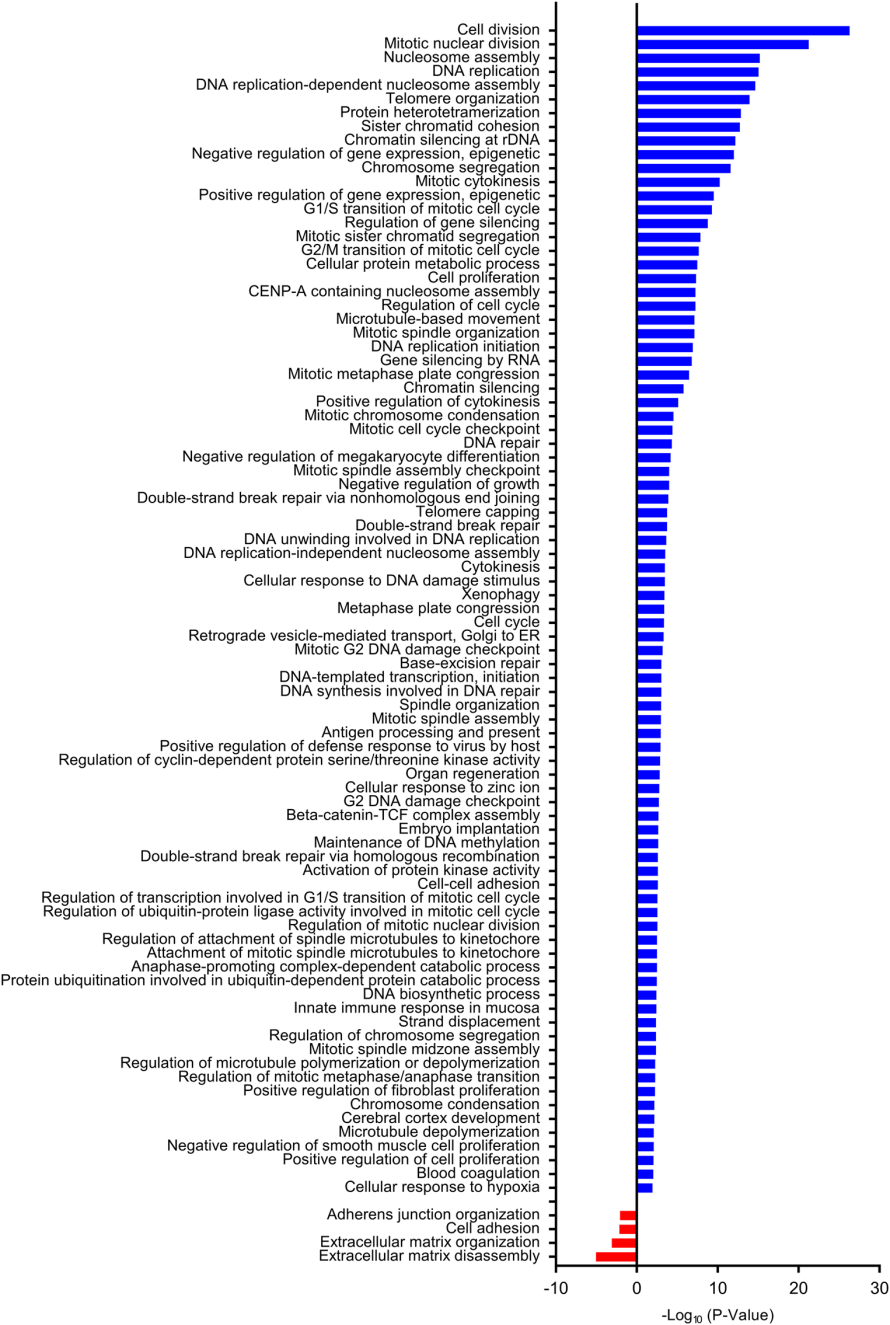
Enrichment analyses of Gene Ontology terms in cultured STAG2 Ser327Asn fibroblast. Bar plots represent the statistical significance of the enrichment (−log_10_(*p*-value)) in STAG2 Ser327Asn fibroblasts. Expression of genes in cell division, mitosis, DNA replication, and DNA damage repair pathways, shown in *blue bars*, was upregulated in the STAG2 Ser327Asn fibroblasts

We next examined the cell cycle profile of normal and patient fibroblasts with flow cytometry. As shown in Fig. 3, the patient fibroblasts had higher percentage of G2/M cells, as compared with two independent lines of normal fibroblasts. Furthermore, microarray analysis revealed the upregulation of the gene expression of cell division, mitotic regulators, and DNA replication factors in patient cells (Fig. 4 and Supplementary Table 2). These results are consistent with a defective cohesin function in transcription regulation. On the other hand, we do not know whether the altered transcription in patient fibroblasts is the cause or consequence of the altered cell cycle profile. Because the normal and patient fibroblasts are not isogenic, we also cannot be certain that the STAG2 mutation is the sole cause of the cell cycle and transcriptional changes.

**Fig. 5:**
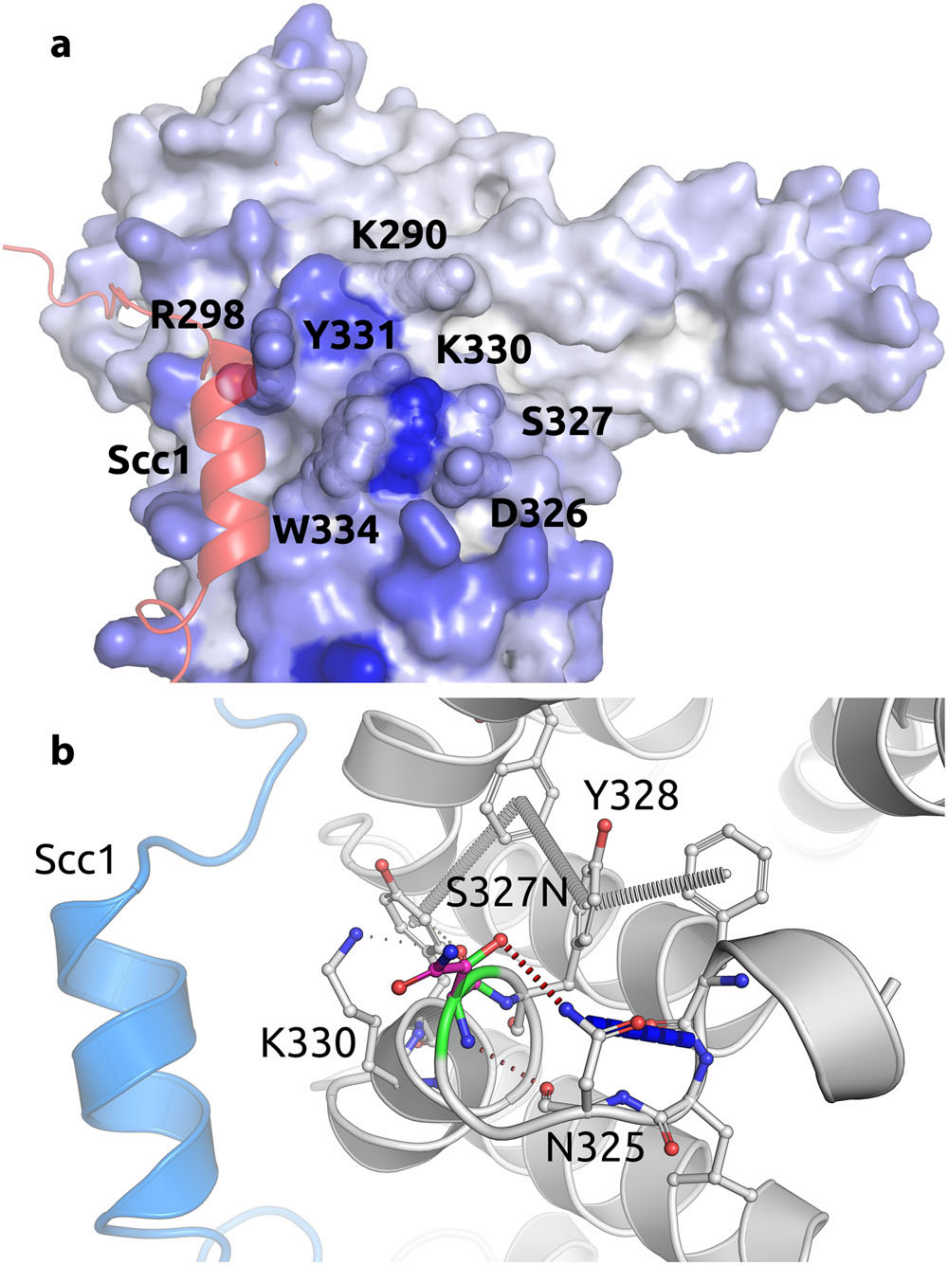
STAG2 residues predicted to be putative interacting sites for protein–protein interfaces based on consensus classifier. **a** Residue colors based on Spider score^47^ from *white to blue*, with residues closer to *blue* with the greater the likelihood to be involved in mediating a protein–protein interaction. SCC1 is shown as cartoon in *red*. **b** Non-covalent interactions made by WT residue. Ser327 forms a hydrogen bond with Asn325 that is substituted to a donor-pi interaction with Tyr328 in the mutant model. SCC1 is shown as cartoon in *blue*. The mutant residue is shown with *dark gray* carbons

We then proceeded with structural and functional experiments of STAG2. Serine 327 is highly conserved among metazoan STAG2 proteins, and forms a side-chain to side-chain hydrogen bond with Asparagine 325 (Fig. 5). The mutation p.Ser327Asn would alter these local intramolecular interactions. The intra-helical hydrogen bond originally made with Asn325 along the edge of the groove is instead substituted by a donor-pi interaction with Tyr328 across the groove, changing also the direction of the interaction orthogonally. The increased volume associated with the mutant residue could also alter the local structure. However, these alterations were not predicted to be significantly destabilizing, nor were they predicated to affect conformational flexibility.^30, 31^

**Fig. 6:**
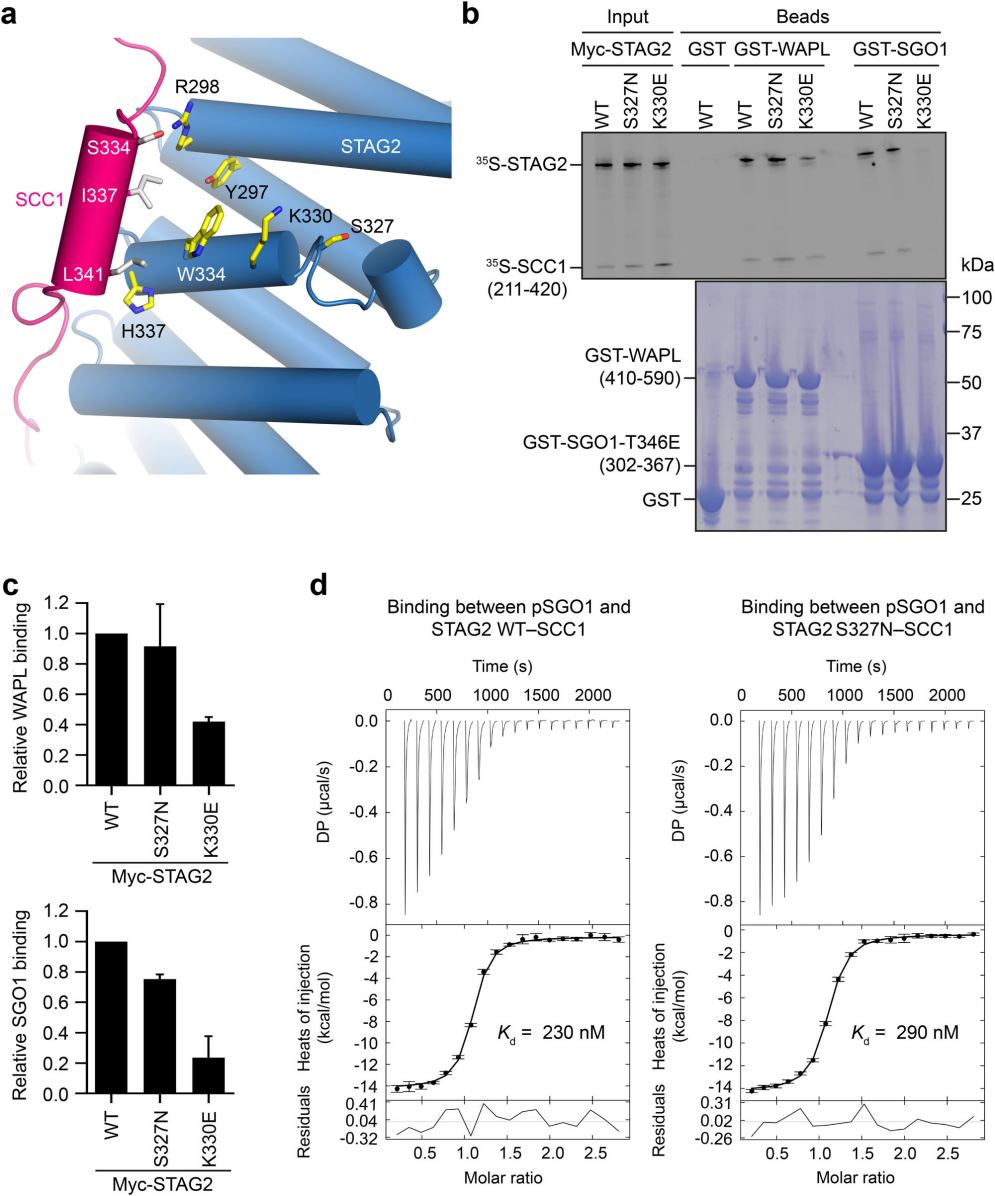
STAG2 p.Ser327Asn retains binding to WAPL and SGO1 in vitro. **a** Cartoon diagram of the crystal structure of human STAG2–SCC1, with Ser327 and neighboring residues shown in *sticks*. **b** In vitro binding of STAG2–SCC1 to GST-WAPL or GST-Sgo1. GST was used as the negative control. *Top* and *bottom* panels show the autoradiograph and Coomassie staining of the binding reactions, respectively. **c** Quantification of the relative WAPL-binding and SGO1-binding activities of STAG2–SCC1 WT, p.Ser327Asn (Ser327Asn), and Lys330Glu (K330E) (normalized to WT) in **b**. Mean ± SD, *n* = 3 independent experiments. For WAPL binding, WT vs. S327N, *p* = 0.6271, not significant; WT vs. K330E, *p* < 0.0001. For SGO1 binding, WT vs. S327N, *p* = 0.0002, *p* < 0.05; WT vs. K330E, *p* = 0.0007, *p* < 0.05. All *p-*values were based on *t*-test. **d** ITC graphs of the binding between pSGO1 and SA2–SCC1 complexes (WT and p.Ser327Asn), with the dissociation constants (*K*_d_) indicated

Ser327 is located within a conserved patch 13 Å away from the extensive protein–protein interface of the STAG2–SCC1 complex. This conserved site that is formed by residues Trp334, Tyr331, Lys330, Asp326, Lys290, Arg298 has been shown to be critical for the binding of STAG2 to the regulators SGO1 and WAPL^23^ (Figs 5, 6a). Analysis of the effects of the mutation on the binding affinity of the partner proteins was evaluated using mCSM-PPI.^32^ This indicated that p.Ser327Asn was likely to have only a minimal disruptive effect on the binding affinities of SGO1, SCC1, and WAPL.

However, due to the close proximity of Ser327 to the SGO1 and WAPL-binding residues, we still tested whether the STAG2 p.Ser327Asn mutant was defective in SGO1 or WAPL binding in vitro. As shown previously, mutation of Lys330, a residue adjacent to Ser327, greatly diminished STAG2 binding to both WAPL and a phospho-mimicking mutant of SGO1 (Fig. 6b, c). By contrast, we did not observe substantial differences between STAG2 wild-type (WT) and p.Ser327Asn in binding to WAPL or SGO1. Quantification of multiple binding reactions revealed that the p.Ser327Asn mutation slightly reduced SGO1 binding (Fig. 6c). As determined by isothermal titration calorimetry (ITC), a synthetic phospho-SGO1 peptide bound to purified STAG2–SCC1 WT and p.Ser327Asn with equilibrium dissociation constants of 230 nM and 290 nM, respectively (Fig. 6d). Thus, the p.Ser327Asn mutation does not greatly affect the binding of STAG2 to WAPL or SGO1 in vitro, consistent with the in silico predictions.

**Fig. 7:**
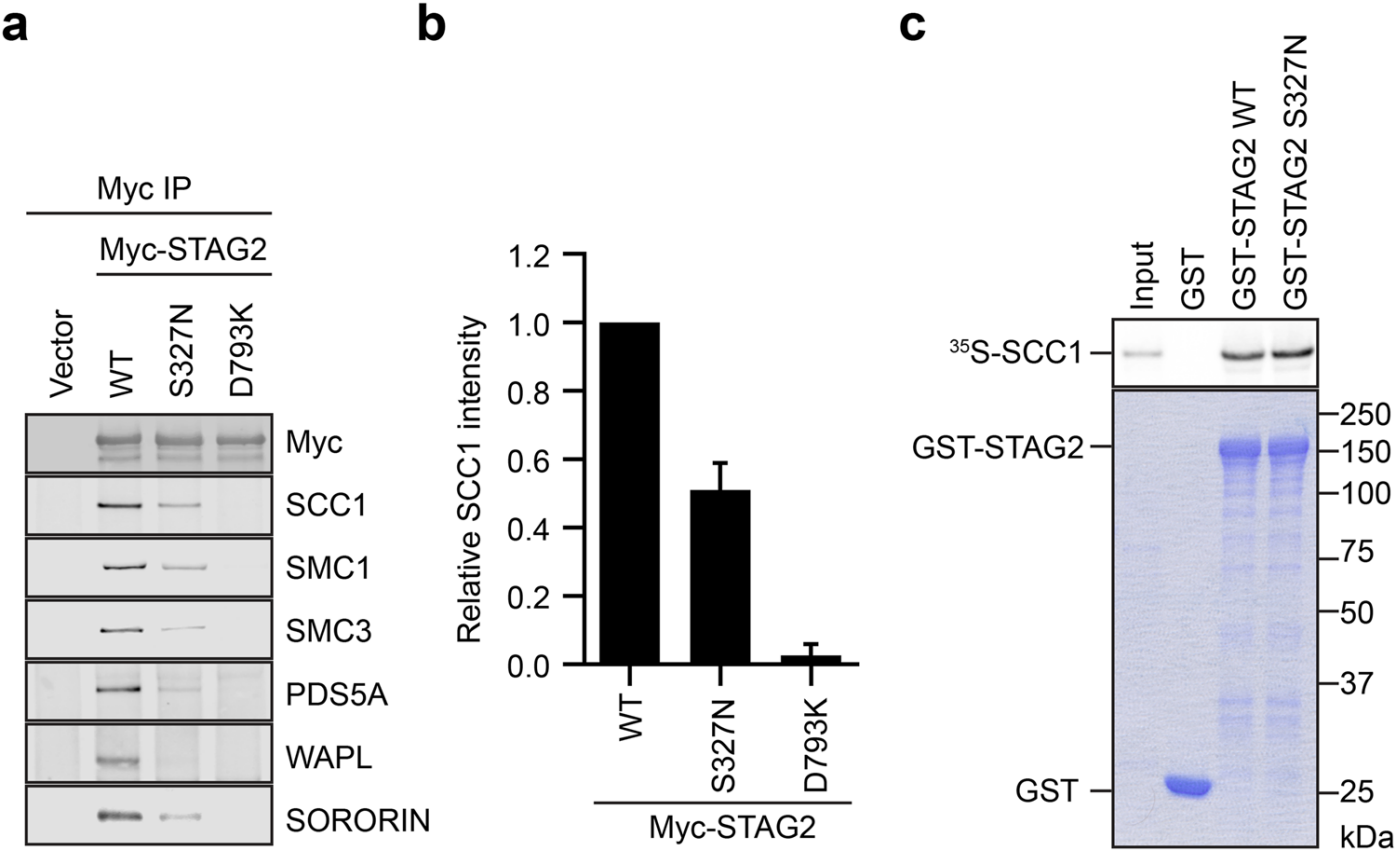
STAG2 p.Ser327Asn (S327N) is defective in binding to cohesin and regulators in human cells. **a** Anti-Myc immunoprecipitates from HeLa cells transfected with the indicated plasmids were blotted with the indicated antibodies. **b** Quantification of the relative SCC1 intensities in blots in **a**. Mean ± SD, *n* = 3 independent experiments. WT vs. S327N, *p* = 0.0004, *p* < 0.05; WT vs. D793K, *p* < 0.0001 (*t*-test). **c** In vitro binding between ^35^S-SCC1 and GST-STAG2 proteins. GST was used as the negative control. *Top* and *bottom* panels show the autoradiograph and Coomassie staining of the binding reaction, respectively

We next examined the effect of the p.Ser327Asn mutation on STAG2 binding to other cohesin subunits and regulators in human cells. Strikingly, in HeLa cells, the p.Ser327Asn mutation reduced Myc-STAG2 binding to the endogenous SCC1, SMC1, and SMC3 by about 50%, and also greatly reduced STAG2 binding to other cohesin regulators, including PDS5A, WAPL, and SORORIN (Fig. 7a, b). The SCC1-binding-deficient STAG2 p.Asp793Lys mutant was included as a control. Because SCC1 is the direct binding partner of STAG2, the defective binding of STAG2 to other cohesin subunits is likely an indirect consequence of defective SCC1 binding, although we cannot rule out the possibility that the p.Ser327Asn mutation also directly affects the binding of some of these components. Because SGO1 only binds to the STAG2–SCC1 complex, not to STAG2 alone,^23^ the defective SCC1 binding by STAG2 p.Ser327Asn strongly suggests that SGO1 binding to STAG2 p.Ser327Asn might also be defective. Therefore, the p.Ser327Asn mutation has a greater effect on STAG2 binding to SCC1 and other cohesin complexes in human cells, as compared with in vitro. We wondered whether the use of an SCC1 fragment in our in vitro binding assay was the underlying cause of this difference. However, purified recombinant GST-STAG2 WT and p.Ser327Asn proteins bound with equal efficiency to full-length SCC1 in vitro (Fig. 7c), ruling out this possibility. On the other hand, due to the poor proliferation properties of the normal control fibroblasts, we cannot obtain enough material to perform the IP-Western experiments to compare the endogenous STAG2-SCC1 interaction in normal and patient cells. Furthermore, because the normal and patient cells are not isogenic, experiments with these cells might not be definitive. Future experiments using isogenic cell lines containing STAG2 WT or p.Ser327Asn are needed to definitively test whether the STAG2 mutation indeed alters cohesin function, and, if so, by what mechanism.

## Discussion

The American College of Medical Genetics (ACMG) and the Association of Molecular Pathology (AMP) have issued rules for the pathogenicity classification of DNA variants.^33^ They recommend that to classify a variant as pathogenic, two or more strong criteria for pathogenicity should be met. In our case, there are three lines of evidence that indicate that the p.Ser327Asn (c.980 G > A) variant is pathogenic and that it is indeed causally related to the *STAG2* phenotypes. First, there is perfect cosegregation of the affected or normal phenotype with, respectively, the presence or absence of the mutation in 17 individuals of the pedigree, as demonstrated by molecular studies (Fig. 1a). Thus, under an X-linked model, the probability that the observed variant-affected status would have occurred by chance rather than by cosegregation, is *N* = (1/2),^17^ i.e., 1/131,072 = 0.0000076.^34^ This very low probability (which corresponds to a logarithm of the odds (LOD) score of 5.12) formally represents strong evidence of pathogenicity according to the proposed modification of the ACMG-AMP pathogenicity classification.^34^ The second line of evidence for pathogenicity is that the patient-derived cells have altered cell cycle and transcriptional profiles, consistent with a defective cohesin function in transcription. Third, with ectoptically expressed proteins, we have demonstrated a defective binding of STAG2 p.Ser327Asn to SCC1 in cultured human cells, which suggests that this mutation might reduce the level of intact cohesin in patient cells. Given the important roles of cohesin in myriad processes, this defect suffices to explain the abnormal phenotypes of the patients harboring the STAG2 p.Ser327Asn mutation. Because we did not observe strong cohesion defects, we propose that altered transcriptional programs as a result of lower concentrations of STAG2-containing cohesin likely underlie the patient phenotype.

Casanova et al.^35^ called attention to the fact that 49 of the 232 monogenic etiologies (21%) of human primary immunodeficiencies were initially reported in single patients. Although our study is based on a single family, it is a large family with six affected individuals and a large LOD score (5.12). This serves as convincing evidence for a causal relationship between the STAG2 p.Ser327Asn mutation and the phenotype of intellectual deficiency, short stature, and other abnormalities observed in our patients, thus defining a new cohesinopathy.

Because we cannot directly verify that the STAG2 p.Ser327Asn mutation weakens the endogenous STAG2–SCC1 interaction in patient cells, it remains a formal possiblity that the defective interaction seen with overexpressed proteins might not reflect the true situation in patient cells. Given the complicated nature of human diseases, we cannot completely rule out the contribution of other factors to the observed phenotypes. At present, we do not yet fully understand why the STAG2 p.Ser327Asn mutation reduces SCC1 binding in human cells, but not in vitro. In the STAG2–SCC1 structure, STAG2 and SCC1 interact through an extensive interface. Numerous STAG2 mutations targeting residues directly located at the protein–protein interface, including Tyr297Ala, Arg298Glu, Trp334Ala, however, do not affect STAG2 binding to SCC1^23^ (Fig. 6a). In fact, among tens of mutants examined previously, only one mutation Asp793Lys targeting a residue in the C-terminal region of STAG2 reduces SCC1 binding. Because Ser327 does not lie at the STAG2–SCC1 interface, it is highly unlikely that it directly contributes to SCC1 binding, consistent with a lack of effect of p.Ser327Asn on SCC1 binding in vitro. However, the highly conserved nature of Ser327 suggests that it most likely plays an important role in a key protein–protein interaction, consistent with the stronger evolutionary restraints on this residue compared with other surface residues.^36^

Assuming that the defective binding of STAG2 p.Ser327Asn to SCC1 in human cells is not caused by trivial technical reasons (e.g., the use of different tags etc.), we hypothesize that this defective binding might be caused by indirect mechanisms. For example, other proteins in human cells might negatively regulate the STAG2–SCC1 interaction, and the p.Ser327Asn mutation strengthens STAG2 binding to the putative inhibitors. Alternatively, STAG2 might transiently interact with SCC1 through a different interface directly involving p.Ser327Asn during the ATPase cycle of the intact cohesin in human cells. High-resolution structures of intact cohesin in various nucleotide states are required to test the second possibility. In any case, our study highlights the importance of human genetics in providing penetrating insight into the assembly of a core chromosome regulator.

## Methods

### Patients

Patients were ascertained through GENE—Núcleo de Genética Médica de Minas Gerais, Belo Horizonte, Brazil. Informed consent was obtained according to current ethical and legal guidelines. The Research Ethics Committee of the Hospital das Clínicas of Universidade Federal de Minas Gerais approved the study protocol with number CAEE 22487913.4.0000.5149 and the National Research Ethics Committee with number 778.728. The study was conducted in accordance with the Declaration of Helsinki.

### Exome sequencing and analysis

Only the proband was subjected to WES, which was performed by the Centre for Applied Genomics, Hospital for Sick Children, Toronto, Canada, using the Ion AmpliSeq Exome Kit (Life Technologies) and the Ion Torrent Proton Sequencer on an Ion PI Template OT2 200 Kit V2 (Life Technologies). All data were aligned to the hg19/GRCh37 reference genome and quality trimmed via Ion Torrent Suite Version 4.0.2 (Life Technologies). Variant calling was performed also using the Ion Torrent Suite and variants were annotated for functional effect by SnpEff 3.1. The average coverage was 145.6-fold, with 93.58% of the target bases being covered at least at 20× and with 59.35% being covered at least at 100×. Variants were narrowed down against databases such as 1000 Genomes Phase 3, NHLBI Exome Sequencing Project (ESP6500), and Single Nucleotide Polymorphism database (dbSNP141) using a software developed in-house called Mendel,MD^37^ and the ENLIS Genome Research software (Enlis Genomics). We further used the online software Exome Walker (http://compbio.charite.de/ExomeWalker/ExomeWalker?id=11), which prioritizes variants of whole exome data by random-walk analysis of protein–protein interactions. To analyze the impact of the candidate variants the software Alamut Visual version 2.6.0 (Interactive Biosoftware) was used, which showed alignment of orthologues genes and includes several protein-function prediction tools: SIFT, PolyPhen-2, and Mutation Taster.

### Allele-specific PCR and Sanger sequencing

The presence of the c.980 G > A variant in *STAG2* gene in the proband and his relatives was confirmed using allele-specific PCR and Sanger sequencing. Allele-specific PCR was achieved by synthesizing long primers that differed on their 3′ extremity, where the mutation was located. The primers were destabilized by introducing different mismatches in the base adjacent to the 3′ extremity, indicated by an underline in the sequences shown. The final sequences were: WT_forward—5′-ATGAAGATGTATAGTGATGCCTTTCTTAATGACCG-3′; c.980 G > A_forward—5′-GATGAAGATGTATAGTGATGCCTTTCTTAATGACTA-3′; Reverse—5′-TGTCACCAGGTATACATACTGGGAGATAACTCATG-3′.

Sanger sequencing was achieved by standard methods using the BigDye Terminator v3.1 Cycle Sequencing Kit (Applied Biosystems^®^) and the Applied Biosystems (ABI) 3130 Genetic Analyzer. Sequencing data was analyzed using the software Sequencher version 4.1.4 (Gene Code).

### Sister chromatid cohesion assay

Chromosome preparations from the patient and controls were obtained from lymphocyte cultures of peripheral blood using standard procedures and Giemsa staining (Merck). Metaphase chromosome spreads from the patient and four controls were analyzed. The cells were analyzed under a NIKON E-400 photomicroscope with a 100×/1.30 Plan Fluor oil-immersion objective equipped with a CCD camera model ER-3339 (Applied Imaging Corp.) and the images were processed with the CytoVisionTM version 2.8 software (Applied Imaging Corp.). A total of 100 metaphases were analyzed per cell line and the quantity of cells with chromosome arms open (chromosome arms showed a clear separation) and closed (chromosome arms had no separation) were scored.

### Primary human fibroblast cultures

Patient, male and female control skin biopsies (*n* = 3) were minced into small pieces with a sterile scalpel in a biological safety cabinet. The explants were left overnight in Dulbecco’s modified Eagle’s medium-high glucose (Sigma-Aldrich) supplemented with 5 mM sodium bicarbonate (Cinética Química Ltda), penicillin (Sigma-Aldrich, 100 units/mL), streptomycin (Sigma-Aldrich, 0.1 mg/mL), amphotericyn B (Sigma-Aldrich, 0.25 μg/mL), gentamicin (Schering-Plough, 60 mg/l), and 10% of fetal bovine serum (FBS) (Cripion Biotecnologia Ltda). Supplemented medium was replaced twice a week. The explants were removed when roughly 90% monolayer confluence was reached, and the cells were subcultured 1:3 until confluence. The cells were frozen at passages 3–4 for subsequent experiments. After thawing, the cells were used in passages 5–9.

### Immunofluorescence

Fibroblasts were seeded at 1 × 10^5^ cells per well on 22 × 22 mm coverslips placed in 6-well plates containing DMEM supplemented with antibiotics and 10% FBS for 24 h. Cells on the coverslips were fixed with 4% paraformaldehyde, and permeabilized with phosphate buffered saline (PBS) containing 0.1% Triton X-100 (Sigma-Aldrich). Slides were incubated with anti-STAG2 (J-12) antibody (Santa Cruz Biotechnology, 1:100) overnight at 4 °C, followed by secondary detection using Alexa 488–conjugated secondary antibody (Life Technologies, 1:500), mounting and nuclear counterstaining using Vectashield (Vector Labs) with Hoechst 33258 (Life Technologies). The samples were examined with the inverted Eclipse Ti microscope (Nikon Instruments) with a Nikon S Fluor 40×/1.30 oil-immersion objective and with appropriate filter combinations. Images were acquired with EMCCD LucaEM R (Andor Technology) using NIS-elements advanced research software (Nikon Instruments). This experiment was performed in triplicate.

### Western blotting

An amount of 1.5 × 10^5^ cells per well were seeded in 6-well plates. The proteins were collected directly from the culture plate wells after treating the cells with lysis buffer followed by protein separation in 10% SDS-PAGE. Gel samples were transferred to polyvinylidene difluoride membrane (GE Healthcare Life Sciences) using standard procedures. Western blot analyses were performed using mouse monoclonal STAG2 antibody (J-12) (Santa Cruz Biotechnology, 1:1000) as primary antibody and anti-mouse IgG (whole molecule)—peroxidase (Sigma-Aldrich, 1:3000) as secondary antibody. The Western blot membrane was mild stripped and re-probed with monoclonal anti-alpha-tubulin antibody clone 5-1-2 (Sigma-Aldrich, 1:10,000) to control for even loading of protein amounts. The membranes were incubated with primary antibodies at 4 °C overnight, washed and incubated with secondary antibodies for 1 h at room temperature. Chemiluminescent detection was performed using Immobilon Western Chemiluminescent HRP Substrate (Millipore), according to the manufacturer’s protocols. Three independent experiments were performed in triplicate.

### Flow cytometry

Fibroblasts were collected with trypsinization and fixed in 70% ice-cold ethanol overnight. After being washed with PBS, cells were permeabilized with PBS containing 0.25% Triton X-100 on ice for 5 min. Cells were then incubated with MPM2 antibody in PBS containing 1% BSA for 3 h at room temperature. After being washed with PBS containing 1% BSA, cells were incubated with a fluorescent secondary antibody for 30 min at room temperature. After being washed with PBS once, cells were resuspended in PBS containing 0.1% Triton X-100, RNase A and propidium iodide, and then analyzed with a flow cytometer. Data were processed with FlowJo. Only DNA contents were shown in the histograms.

### Microarray analysis

Total RNA was extracted from cultured STAG2 WT or Ser327Asn fibroblasts and analyzed by GeneChip Human Transcriptome Array 2.0, which was designed with approximately ten probes per exon and four probes per exon–exon splice junction. Gene expression levels were compared between WT and Ser327Asn samples. Gene ontology enrichment analysis were performed on the genes with more than four-fold alterations between WT and Ser327Asn using the DAVID Bioinformatics Database (http://david.abcc.ncifcrf.gov).

### Computational protein structure analysis for functional studies

Mutations can affect or disrupt protein structure and function. Computational structure-based approaches have been shown to be invaluable tools to unravel the molecular mechanism of mutations giving rise to a phenotype.^38–41^

In order to assess the structural effects of the mutation, the available X-ray crystal structure of the human STAG2 in complex with SCC1 was used (PDB ID’s: 4PJU, 4PJW, and 4PK7). Based on these structures, models of complexes of STAG2 with the other cohesin subunits SGO1 and WAPL were generated using Modeller^42^ and MacroModel (Schrodinger, New York, NY).

The effects of the mutations on protein stability and affinity for its partners were assessed in this work using two methods: DUET^30^ and mCSM-PPI.^32^ They represent a class of novel machine-learning methods that extract patterns from graph representations of the three-dimensional residue environment structure in order to quantitatively predict the effects of missense mutations on protein stability,^30, 32^ protein–protein interactions,^32, 43^ protein–nucleic acid interactions,^30^ protein small-molecule interactions,^44, 45, 46^ and protein–metal ion interactions.^31^

### Functional studies—antibodies

The anti-WAPL and anti-SORORIN antibodies were generated against human WAPL_601–1190_ and SORORIN_91–252_, respectively. The following antibodies were purchased from the indicated commercial sources: anti-Myc (Roche, 11667203001), anti-SCC1 (Bethyl Laboratories, A300-080A), anti-SMC1 (Bethyl Laboratories, A300-055A), anti-SMC3 (Bethyl Laboratories, A300-060A), and anti-PDS5A (Bethyl Laboratories, A300-089A).

### Mammalian cell culture, transfection, and immunoprecipitation for functional studies

HeLa Tet-On cells were grown in DMEM (Invitrogen) supplemented with 10% FBS and 2 mM L-glutamine. When cells reached a confluency of 50%, plasmid transfection was performed using the Effectene reagent (Qiagen), according to the manufacturer’s protocols. HeLa Tet-On cells were transfected with a pCS2 plasmid encoding codon-optimized human STAG2 WT or mutants with a Myc tag at the N-terminus. Cells were trypsinized and harvested at 30 h after transfection. For immunoprecipitation, the anti-Myc antibody was coupled to Affi-Prep Protein A beads (Bio-Rad) at a concentration of 1 mg/ml. Cells were resuspended in the lysis buffer containing 25 mM Tris-HCl (pH 7.7), 50 mM NaCl, 0.1% (v/v) Nonidet P-40, 2 mM MgCl_2_, 10% (v/v) glycerol, 5 mM NaF, 0.3 mM Na_3_VO_4_, 10 mM β-glycerophosphate, 1 mM DTT, protease inhibitor mixture (Roche), and 50 units/ml Turbo Nuclease (Accelagen). The cell suspension was passed through a 27 G × 1/2 in (0.4 × 13 mm) needle 20 times. Cell lysates were then incubated on ice for 1 h followed with a 10-min incubation at 37 °C. Cell lysates were centrifuged at 4 °C at 20,817*g* for 20 min. The supernatants were incubated with the anti-Myc antibody beads for 3 h at 4 °C. The beads were then washed three times with the lysis buffer containing 200 mM NaCl. Proteins bound to beads were dissolved in SDS sample buffer, separated by SDS-PAGE, and blotted with the appropriate antibodies.

### Protein expression and purification for functional studies

The cDNAs of human SCC1_281–394_, full-length STAG2 WT or Ser327Asn, WAPL_410–590_, and SGO1_302–367_ T346E were cloned into the pGEX-6p1 vector. The cDNA of human STAG2_80–930_ was cloned into the pET28a vector. For the in vitro binding assays, recombinant GST fusion proteins of STAG2 (WT and Ser327Asn), WAPL_410–590_, or SGO1_302–367_ Thr346Glu were expressed in *E. coli* and purified with the Glutathione Sepharose 4B resin (GE Healthcare). All fragments of cohesin subunits and regulators contained the miminal functional domains required for their interactions.^23^ Cell pellets were resuspended in the lysis buffer (50 mM Tris-HCl (pH 7.7), 150 mM NaCl, 0.05% (v/v) Triton X-100, 5% (v/v) glycerol, and 1 mM DTT). After sonication and centrifugation, the supernatant was applied to the Glutathione Sepharose 4B beads that had been equilibrated with the lysis buffer, and incubated at 4 °C for 2 h. Proteins bound to beads were washed with the lysis buffer, and then eluted with the elution buffer (50 mM Tris-HCl (pH 8.0), 15 mM reduced glutathione (Sigma), and 1 mM DTT). Reduced glutathione was removed using the PD-10 column (GE Healthcare). For the ITC assay, SCC1_281–394_ in the pGEX-6p1 vector and STAG2_80–930_ in the pET28a vector were co-transformed into *E. coli*. The STAG2_80–930_–SCC1_281–394_ complex was pulled down with the Glutathione Sepharose 4B beads (GE Healthcare), incubated with the PreScission protease to remove the GST tag, and further purified with ion exchange and size exclusion columns.

### Isothermal titration calorimetry

ITC was performed with a MicroCal iTC200 (GE Healthcare) at 20 °C. Calorimetric measurements were performed with the STAG2_80–930_–SCC1_281–394_ complex and a synthetic phospho-Thr346 SGO1 peptide containing residues 313–353 of human SGO1. A 20 μM of STAG2_80–930_–SCC1_281–394_ complex in a buffer containing 20 mM Tris-HCl (pH 7.7), 200 mM NaCl, and 5 mM TCEP was added to the calorimeter cell, and titrated with 300 μM of the pSGO1 peptide in the same buffer. Binding parameters were calculated with the NITPIC software.

### In vitro binding assays for functional studies

The cDNAs encoding full-length human SCC1, SCC1_211–420_, or STAG2 were cloned into the pCS2-Myc vector. The pCS2-SCC1-Myc vector was added to the TNT Quick Coupled Transcription Translation System (Promega) and incubated in the presence of ^35^S-methionine at 30 °C for 90 min to produce ^35^S-labeled SCC1-Myc. The pCS2-SCC1_211–420_-Myc and pCS2-Myc-STAG2 vectors were added together to the TNT Quick Coupled Transcription Translation System and incubated in the presence of ^35^S-methionine at 30 °C for 90 min to produce the ^35^S-labeled STAG2–SCC1_211–420_ complex. To assay the binding between STAG2 and SCC1, Glutathione Sepharose 4B beads (GE Healthcare) bound to GST-STAG2 WT or p.Ser327Asn were incubated with ^35^S-SCC1-Myc at 4 °C for 2 h, and then washed four times with TBS containing 0.05% Tween 20. To assay the binding of SGO1 or WAPL to STAG2–SCC1, Glutathione Sepharose 4B beads (GE Healthcare) bound to GST-SGO1_302–367_ T346E or GST-WAPL_410–590_ were incubated with the ^35^S-STAG2–Scc1_211–420_ complex at 4 °C for 2 h, and then washed four times with TBS containing 0.05% Tween 20. In all cases, beads bound to GST were used as the negative control. The bound proteins were separated by SDS-PAGE and analyzed with a phosphoimager. Intensities of bound proteins were quantified with ImageJ.

## Electronic supplementary material

References for Supplemental Material

Legend for Fig S1

Supplementary Table 1

Supplementary Table 2

Supplementary Figure S1
