# Supplementary material for: Familial STAG2 germline mutation defines a new human cohesinopathy
Source: NPJ Genom Med. 2017 Mar 20;2:7. doi: 10.1038/s41525-017-0009-4 (PMC5677968; doi:10.1038/s41525-017-0009-4)
Supplement: Supplementary file 1 — References for Supplemental Material [file 41525_2017_9_MOESM1_ESM.docx]

**Supplementary References for Table S1**

## Jones KL (ed) Smith's Recognizable Paterns of Human Malformation Edn.6th (Elsevier Saunders, Philadelphia, USA, 2006).

Boat T.F. & Wu J.T. *Mental Disorders and Disabilities Among Low-Income Children*. 472 pp (The National Academies Press, Washington, USA, 2015).
