# Supplementary material for: Familial STAG2 germline mutation defines a new human cohesinopathy
Source: NPJ Genom Med. 2017 Mar 20;2:7. doi: 10.1038/s41525-017-0009-4 (PMC5677968; doi:10.1038/s41525-017-0009-4)
Supplement: Supplementary file 2 — Legend for Fig S1 [file 41525_2017_9_MOESM2_ESM.docx]

**Figure S1 | Cartoon of the structure of the cohesin supramolecular assembly, of the known cohesinopathies (including described in this study) and of the genes that when mutated cause cohesinopathies.** The cohesinopathies fall into four phenotypes: Roberts Syndrome/SC-Phocomelia phenotype, caused by pathogenic mutations in *ESCO2* (number 1); Cornelia de Lange Syndrome, which can be caused by pathogenic mutations in *NIPBL* (CdLS1; number 2), *SMC1* (CdLS2; number 3), *SMC3* (CdLS3, number 4), *SCC1* (CdLS4, number 5) and *HDAC8* (CdLS5, number 6); Chronic Atrial and Intestinal Dysrhythmia (CAID) caused by pathogenic mutations in *SGO1* (number 7) and *STAG2*-related Syndromic X-Linked Intellectual Deficiency described here (number 8).
