# Supplementary figures and images for: Familial STAG2 germline mutation defines a new human cohesinopathy
Source: NPJ Genom Med. 2017 Mar 20;2:7. doi: 10.1038/s41525-017-0009-4 (PMC5677968; doi:10.1038/s41525-017-0009-4)

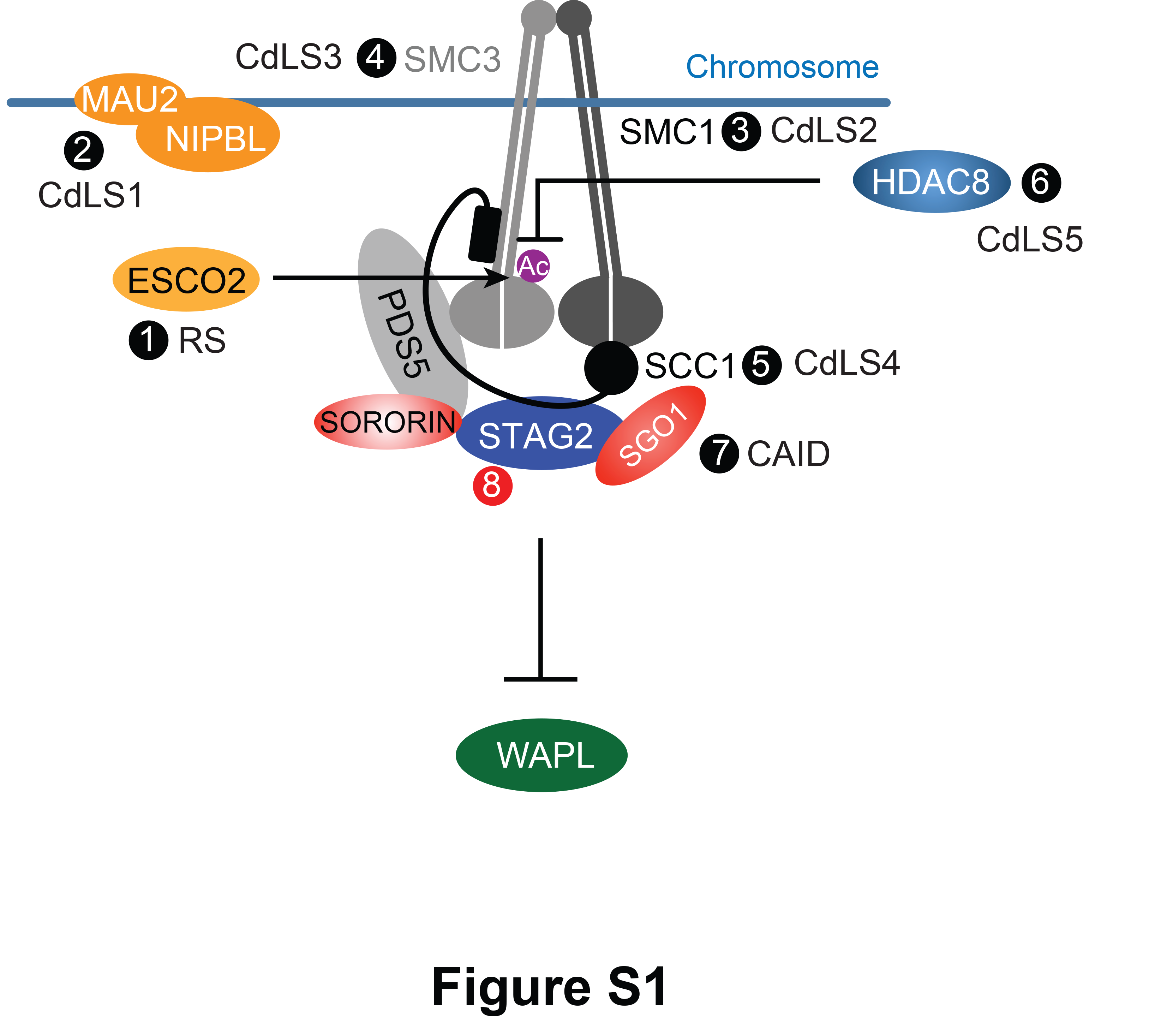

Supplement: Supplementary file 5 — Supplementary Figure S1 [file 41525_2017_9_MOESM5_ESM.tif]
